# Childhood pre-B cell acute lymphoblastic leukemia with translocation t(1;19)(q21.1;p13.3) and two additional chromosomal aberrations involving chromosomes 1, 6, and 13: a case report

**DOI:** 10.1186/s13256-017-1251-1

**Published:** 2017-04-07

**Authors:** Abdulsamad Wafa, Manar As’sad, Thomas Liehr, Abdulmunim Aljapawe, Walid Al Achkar

**Affiliations:** 1 Molecular Biology and Biotechnology Department, Human Genetics Div., Atomic Energy Commission of Syria, P.O. Box 6091, Damascus, Syria; 2 Jena University Hospital, Institute of Human Genetics, Kollegiengasse 10, D-07743 Jena, Germany; 3 Molecular Biology and Biotechnology Department, Mammalians Biology Div., Flow-cytometry Lab., Atomic Energy Commission of Syria, P.O. Box 6091, Damascus, Syria

**Keywords:** Acute lymphoblastic leukemia (ALL), t(1;19), *TCF3-PBX1*, Cytogenetics, Fluorescence *in situ* hybridization (FISH), Prognostic factors

## Abstract

**Background:**

The translocation t(1;19)(q23;p13), which results in the *TCF3-PBX1* chimeric gene, is one of the most frequent rearrangements observed in B cell acute lymphoblastic leukemia. It appears in both adult and pediatric patients with B cell acute lymphoblastic leukemia at an overall frequency of 3 to 5%. Most cases of pre-B cell acute lymphoblastic leukemia carrying the translocation t(1;19) have a typical immunophenotype with homogeneous expression of CD19, CD10, CD9, complete absence of CD34, and at least diminished CD20. Moreover, the translocation t(1;19) correlates with known clinical high risk factors, such as elevated white blood cell count, high serum lactate dehydrogenase levels, and central nervous system involvement; early reports indicated that patients with translocation t(1;19) had a poor outcome under standard treatment.

**Case presentation:**

We report the case of a 15-year-old Syrian boy with pre-B cell acute lymphoblastic leukemia with abnormal karyotype with a der(19)t(1;19)(q21.1;p13.3) and two yet unreported chromosomal aberrations: an interstitial deletion 6q12 to 6q26 and a der(13)t(1;13)(q21.1;p13). According to the literature, cases who are translocation t(1;19)-positive have a significantly higher incidence of central nervous system relapse than patients with acute lymphoblastic leukemia without the translocation. Of interest, central nervous system involvement was also seen in our patient.

**Conclusions:**

To the best of our knowledge, this is the first case of childhood pre-B cell acute lymphoblastic leukemia with an unbalanced translocation t(1;19) with two additional chromosomal aberrations, del(6)(q12q26) and t(1;13)(q21.3;p13), which seem to be recurrent and could influence clinical outcome. Also the present case confirms the impact of the translocation t(1;19) on central nervous system relapse, which should be studied for underlying mechanisms in future.

## Background

Translocation t(1;19)(q23;p13) is a repeatedly but overall rarely observed rearrangement in B cell acute lymphoblastic leukemia (B-ALL), and can be found in both adult and pediatric patients at an overall frequency of 3 to 5% [1, 2]. Most cases of pre-B-ALL carrying the translocation t(1;19) express a typical immunophenotype with homogeneous expression of CD19, CD10, CD9, complete absence of CD34, and reduced expression of CD20 [3]. This translocation can occur in two forms: a balanced or an unbalanced one. The unbalanced form of pre-B-ALL leads to a derivative of chromosome 19 [2]. Both the balanced and the unbalanced translocations t(1;19) result in the fusion of transcription factor 3 (*TCF3*) located in 19p13 with pre-B cell leukemia homeobox 1 (*PBX1*) in 1q23, to form a chimeric gene whose protein product alters, among other cellular processes, cell differentiation arrest [1]. Specifically, the fusion gene encodes for a transcription factor bearing the transactivation domain of *TCF3* and the deoxyribonucleic acid (DNA)-binding domain of *PBX1*, which facilitates genes constitutive activation [4]. In addition, the fusion protein appears to have a dominant negative effect on the wild-type *TCF3* activity. So both increased expression of *PBX1* target genes and reduction in *TCF3* activity are thought to be important in leukemogenesis [5].

Other partner genes of *TCF3* include *ZNF384* (12p13; prognosis unknown), *NOL1* (12p13; prognosis unknown), an unknown partner gene in 13q14 (prognosis unknown), *HLF* (17q22; extremely poor prognosis), and *FB1/TFPT* (19q13.4; prognosis unknown) [6–8]. Moreover, the translocation t(1;19) correlates with known clinical high risk features, such as elevated white blood cell (WBC) count, high serum lactate dehydrogenase (LDH) levels, central nervous system (CNS) involvement, and poor outcome under standard treatment [9].

Here we report the clinical, G-banding, and molecular cytogenetic results obtained in a childhood patient with pre-B-ALL with an unbalanced translocation t(1;19) and two additional chromosomal aberrations.

## Case presentation

On 22 May 2014, a 15-year-old Syrian boy without significant personal or familial history of malignancies presented to Al-Biruni University Hospital with a 1-month history of bleeding gums, fatigue, and pallor. He had no familial history of malignancies and no social and environmental history or exposure to toxins and animals. A physical examination revealed splenomegaly without lymph node involvement. An initial laboratory evaluation of peripheral blood (PB) revealed elevated WBC count of 56×10^9^/l with 94% of blasts cells, anemia (9.1 g/dL), and a thrombocytopenia (123×10^9^/L). His serum LDH value was 862 U/l (normal value up to 480 U/l). He was diagnosed as having pre-B-ALL according to the World Health Organization (WHO) classification. A biopsy of his bone marrow (BM) revealed hypercellular marrow (WBC count 155.3×10^9^/l with 94% of blasts cells).

He was referred (on 25 May 2014) to our Chromosomes Laboratory and Flow-cytometry Laboratory, Molecular Biology and Biotechnology Department, Atomic Energy Commission of Syria, for cytogenetics and flow-cytometric analyses. He could be classified in a high risk group. Thus, he was supplied with German Multicenter Study Group for Adult Acute Lymphoblastic Leukemia (GMALL) protocol treatment after the initial diagnosis for 5 months. Unfortunately, due to the political situation in his home country he was given available drugs such as vincristine 1.4 mg/m^2^, doxorubicin 25 mg/m^2^, and methotrexate 20 mg/m^2^. He responded to that treatment without any infiltrations in his BM; he received platelets and blood transfusions many times; his PB showed pancytopenia and neutropenia. The treatment was stopped before consolidation phase because his BM biopsy evaluation showed morphologic remission (8% blasts left); a cerebrospinal fluid test revealed abnormal cells; he also had a pulmonary infection. Approximately 5.5 months after initial diagnosis he died due to unknown causes while under treatment. No autopsy was performed because he died in his house. His parents agreed with the scientific evaluation of the case and a study was approved by the ethical committee of the Atomic Energy Commission, Damascus, Syria.

**Fig. 1:**
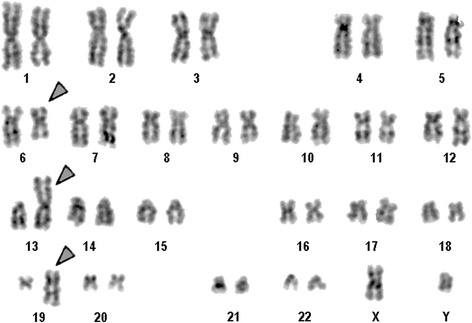
GTG-banding revealed the following karyotype in 6/18 metaphases: 46,XY,del(6)(q?),der(13)t(1;13),der(19)t(1;19)[6]. All derivative chromosomes are marked and highlighted by *arrow heads*

A chromosome analysis on a BM sample using GTG-banding according to standard procedures [10] was performed before his treatment started; it revealed a karyotype of 46,XY,del(6)(q?),der(13)t(1;13),der(19)t(1;19)[6]/46,XY,del(6)(q?),der(19)t(1;19)[5]/47,XY,+i(1)(q?),del(6)(q?),der(19)t(1;19)[1]/46,XY[6] (Fig. 1). Karyotype was described according to the International System for Human Cytogenetic Nomenclature (ISCN) of 2013 [11].

**Fig. 2:**
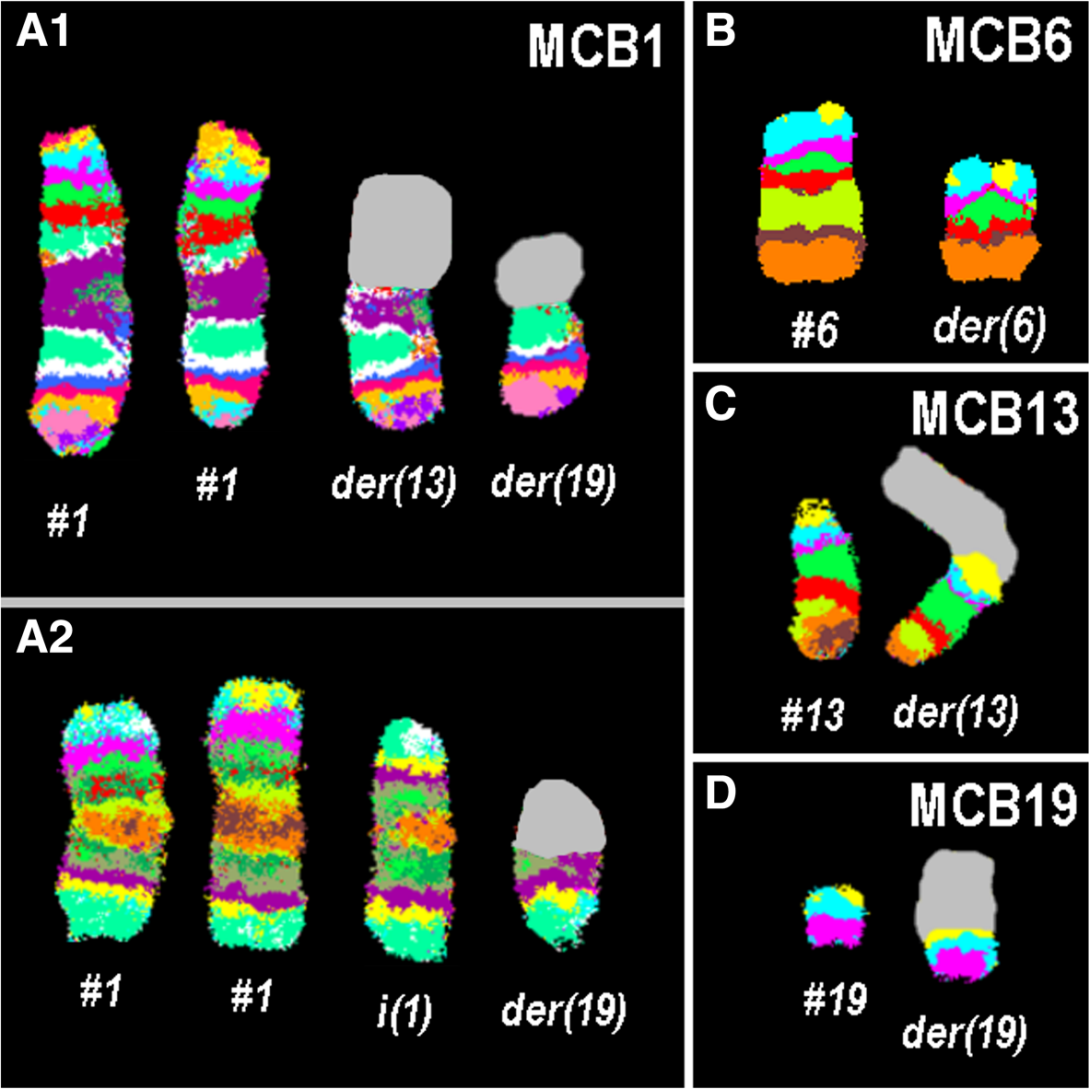
Array-proven multicolor banding results are shown. The normal chromosomes (#) are depicted on the *left side* of each image and the derivative of the four chromosomes on the *right side* of normal chromosomes. The unstained regions when suing chromosome-specific array-proven multicolor banding probe sets on the derivative chromosomes are shown in *gray*. **a** Different pseudocolor depictions for array-proven multicolor banding1 probe set revealed the breakpoints in derivative chromosomes 13, 19 and isochromosome 1q. **b** Array-proven multicolor banding6 uncovered the interstitial deletion in der(6). **c** Array-proven multicolor banding13 revealed that practically the whole short arm of chromosome 13 remained intact in the der(13). **d** The breakpoint on der(19) could be determined by multicolor banding19 probe set. # chromosome, *der* derivative chromosome, *MCB* multicolor banding

Further studies were performed on BM sample using molecular cytogenetics (Fig. 2). We performed dual-color fluorescence *in situ* hybridization (D-FISH) with specific whole chromosome paint (WCP) probes for chromosomes 1, 6, 13, and 19 (MetaSystems, Altlussheim, Germany) [10], which did not provide any information on the cryptic translocations (data not shown), and array-proven high-resolution multicolor banding (aMCB) [12], using probes for the corresponding chromosomes 1, 6, 13, and 19 involved according to GTG-banding (Fig. 2). Reverse transcriptase-polymerase chain reaction (RT-PCR) for *E2A-PBX1* fusion transcripts was performed prior the treatment using specific primers previously described [13], confirmed the presence of the *TCF3*/*PBX1* fusion *(E2A*/*PBX1* transcript; 373-base pair band), most often identified in acute lymphoblastic leukemia (ALL; data not shown). Thus, the following final karyotype prior to treatment was determined using a fluorescence microscope (AxioImager.Z1 mot, Carl Zeiss Ltd, Hertfordshire, UK) equipped with appropriate filter sets to discriminate between a maximum of five fluorochromes plus the counterstain 4',6-diamidino-2-phenylindole (DAPI). Image capture and processing were performed using an ISIS imaging system (MetaSystems): 46,XY,del(6)(q12q26),der(13)t(1;13)(q21.1;p13),der(19)t(1;19)(q21.1;p13.3)[6]/46,XY,del(6)(q12q26),der(19)t(1;19)(q21.1;p13.3)[5]/47,XY,+i(1)(q10),del(6)(q12q26),der(19)t(1;19)(q21.1;p13.3)[1]/46,XY[6].

Immunophenotyping was performed on BM sample using a general panel of fluorescent antibodies against the following antigens typical for different cell lineages and cell types: CD1a, CD2, CD3, CD4, CD5, CD8, CD10, CD11b, CD11c, CD13, CD14, CD15, CD16, CD19, CD20, CD22, CD23, CD32, CD33, CD34, CD38, CD41a, CD45, CD56, CD57, CD64, CD103, CD117, CD123, CD138, CD209, CD235a, and CD243; in addition, antibodies to kappa and lambda light chains, IgD, surface-bound IgM (sIgM), and human leukocyte antigen-antigen D related (HLA-Dr) were tested. All antibodies were purchased from BD Biosciences (San Jose CA, USA). Samples were analyzed on a BD FACSCalibur™ flow cytometer. Autofluorescence, viability, and isotype controls were included. Flow cytometric data acquisition and analysis were conducted by BD Cellquest™ Pro software. Flow cytometric analysis of BM specimen characterized this case as pre-B-ALL according to WHO classifications. The abnormal cell population (94% of tested cells) was positive for CD45^+dim^, CD19^+^, CD10^+^, HLA-DR^+^, and CD79a^+^. This cell population was negative for CD34, CD2, CD7, CD13, CD14, CD15, CD20, CD33, and CD117.

## Discussion

According to the literature, the translocation t(1;19)(q23;p13) is a translocation that is repeatedly but, overall, rarely seen in B-ALL cases; it generates the *TCF3-PBX1* fusion gene [1, 2]. According to Mitelman Database of Chromosome Aberrations in Cancer [14], there are five cases of ALL with translocation t(1;19)(q21;p13), seven such cases with translocation t(1;13) involving short and/or long arms of both chromosomes, five cases of ALL with isochromosome i(1)(q10), and 710 cases of ALLs with del(6)(q) including 21 cases with del(6)(q12) and three cases with del(6)(q26). In addition, the chromosomal bands 1q21, 13p13, and 19p13 are involved in chromosomal rearrangements in 252, 785, and eight cases, respectively [14]. To the best of our knowledge, the present case report is the first one to observe a childhood pre-B-ALL with unbalanced translocation t(1;19)(q21.1;p13.3) associated with two additional chromosomal aberrations del(6)(q12q26) and der(13)t(1;13)(q21.1;p13) [14]. Of interest, one metaphase with an isochromosome 1q was also observed in GTG-banding and found in 1/25 metaphases in fluorescence *in situ* hybridization (FISH) analyses too (Fig. 2). As aforementioned this was also seen in five earlier cases of ALL [14].

The translocation t(1;19)(q23;p13) can be observed in two principal forms [2, 15, 16]. Abnormalities of chromosome 1, such as complete or partial trisomies for the long arm, are known to arise during clonal evolution and can be observed as recurrent in hematologic malignancies [16–18].

The *CKS1B* gene mapped at 1q21 is a member of the highly conserved cyclin kinase subunit 1 (CKS1) protein family that interacts with cyclin-dependent kinases (Cdks) and plays a critical role in cell cycle progression [19]. *CKS1B* overexpression is correlated with low p27 expression and adverse survival in several human malignancies including gastric, colorectal, and oral squamous cell carcinomas [20–22] because of its critical role as a cell cycle regulator and its involvement in various human carcinomas.

This 6q12-22 area contains several genes with a known or suspected tumor-suppressing function including *MAP3K7*, for which a tumor-suppressive role for prostate epithelial cells has recently been demonstrated [23]. *MAP3K7* is ubiquitary expressed and involved in various biological processes such as cell growth, differentiation, and apoptosis. *MAP3K7* exerts these effects by interacting with a number of different signaling pathways and activator molecules [24–27].

In contrast to early reports on patients with translocation t(1;19) reporting a more adverse outcome for patients with balanced translocation compared with patients with an unbalanced translocation t(1;19) [28–30], more recent studies found no difference in event-free survival (EFS) among these two subgroups [30–32]. However, cases who are translocation t(1;19)-positive have a significantly higher incidence of CNS involvement in cases of relapse than patients with ALL without the translocation [30]; the St Jude’s group observed four patients with CNS relapse but no patients with BM relapse among 41 patients with translocation t(1;19) [33]. Also, Moorman *et al*. [34] reported six relapses in 50 patients with translocation t(1;19) of which three relapses involved the CNS. Moreover, CNS disease was found to be more common in patients with T cell acute lymphoblastic leukemia (T-ALL) compared with those with pre-B-ALL [35], and CNS involvement was associated with poor survival. Fielding *et al*. [36] showed as well that the outcome of relapsing patients was very poor, and that patients with T cell disease had only a 5% 5-year survival compared with 8% in those with B-ALL.

Additional cytogenetic abnormalities (ACAs) were observed in 63% (47 out of 2640, which is 1.8%, children diagnosed as having B-ALL), with no significant differences between translocation t(1;19) and/or der(19)t(1;19)-positive cases [37]. The most common ACAs were del(9p), i(9q), del(6q), and del(13q) [16, 20]. None of their 47 patients with translocation t(1;19) demonstrated concomitant aberrations of 13p13 and/or 6q12 to 6q26 regions. Thus, the clinical significance of an unbalanced translocation t(1;19)(q21.1;p13.3) in our patient with a 13p13 translocation and/or del(6)(q12q26) is unclear. According to the literature the presence of ACAs has no significant impact on EFS or overall survival [35]. This observation is in line with findings from other recurrent leukemia-associated translocations, where ACAs seem to have no influence on prognosis [37, 38]. Still, the adverse outcome of the present case may be a hint that there are exceptions to that rule.

Patients with pediatric ALL with WBC counts of more than 50×10^9^/l are considered to be at high risk of relapse and thus receive intensive treatment [39, 40]. In retrospective analysis, patients with hyperleukocytosis (WBC count >50×10^9^/l) were significantly correlated with shorter survival times. The cytogenetic features were closely linked to the WBC and at least partly explain the prognostic value of WBC, although there is evidence that children with similar cytogenetic aberrations may have very different WBCs [41–43].

## Conclusions

Here, we described the presence of two uncommon chromosomal aberrations, that is, del(6)(q12q26) and der(13)t(1;13)(q21.1;p13) in a case of childhood pre-B-ALL with unbalanced translocation t(1;19), which might suggest that special ACAs could have an adverse effect on clinical outcome, in contrast to what is widely discussed at present.

## Abbreviations

ACAs: Additional cytogenetic abnormalities
ALL: Acute lymphoblastic leukemia
aMCB: Array-proven high-resolution multicolor banding
B-ALL: B cell acute lymphoblastic leukemia
BM: Bone marrow
Cdks: Cyclin-dependent kinases
CKS1: Cyclin kinase subunit 1
CNS: Central nervous system
DAPI: 4',6- diamidino-2-phenylindole
D-FISH: Dual-color fluorescence *in situ* hybridization
DNA: Deoxyribonucleic acid
EFS: Event-free survival
FISH: Fluorescence *in situ* hybridization
GMALL: German Multicenter Study Group for Adult Acute Lymphoblastic Leukemia
HLA-Dr: Human leukocyte antigen-antigen D related
ISCN: International System for Human Cytogenetic Nomenclature
LDH: Lactate dehydrogenase
PB: Peripheral blood
*PBX1*: Pre-B cell leukemia homeobox 1
RT-PCR: Reverse transcriptase-polymerase chain reaction
sIgM: Surface-bound IgM
T-ALL: T cell acute lymphoblastic leukemia
*TCF3*: Transcription factor 3
WBC: White blood cell
WCP: Whole chromosome paint
WHO: World Health Organization
